# Exercise Adherence and Effect of Self-Regulatory Behavior Change Techniques in Patients Undergoing Curative Cancer Treatment: Secondary Analysis from the Phys-Can Randomized Controlled Trial

**DOI:** 10.1177/1534735420946834

**Published:** 2020-09-10

**Authors:** Anne-Sophie Mazzoni, Hannah L Brooke, Sveinung Berntsen, Karin Nordin, Ingrid Demmelmaier

**Affiliations:** 1 Department of Public Health and Caring Sciences, Uppsala University, Uppsala, Sweden; 2 Department of Sport Science and Physical Education, University of Agder, Norway

**Keywords:** adherence, adjuvant treatment, behavioral intervention, behavior change support, exercise prescription, oncology, physical activity

## Abstract

**Introduction:**

Adherence to exercise interventions in patients with cancer is often poorly described. Further, it is unclear if self-regulatory behavior change techniques (BCTs) can improve exercise adherence in cancer populations. We aimed to (1) describe exercise adherence in terms of frequency, intensity, time, type (FITT-principles) and dropouts, and (2) determine the effect of specific self-regulatory BCTs on exercise adherence in patients participating in an exercise intervention during curative cancer treatment.

**Methods:**

This study was a secondary analysis using data from a Swedish multicentre RCT. In a 2×2 factorial design, 577 participants recently diagnosed with curable breast, colorectal or prostate cancer were randomized to 6 months of high (HI) or low-to-moderate intensity (LMI) exercise, *with* or *without* self-regulatory BCTs (e.g., goal-setting and self-monitoring). The exercise program included supervised group-based resistance training and home-based endurance training. Exercise adherence (performed training/prescribed training) was assessed using attendance records, training logs and heart rate monitors, and is presented descriptively. Linear regression and logistic regression were used to assess the effect of self-regulatory BCTs on each FITT-principle and dropout rates, according to intention-to-treat.

**Results:**

For resistance training (groups *with* vs *without* self-regulatory BCTs), participants attended on average 52% vs 53% of prescribed sessions, performed 79% vs 76% of prescribed intensity, and 80% vs 77% of prescribed time. They adhered to exercise type in 71% vs 68% of attended sessions. For endurance training (groups *with* vs *without* self-regulatory BCTs), participants performed on average 47% vs 51% of prescribed sessions, 57% vs 62% of prescribed intensity, and 71% vs 72% of prescribed time. They adhered to exercise type in 79% vs 78% of performed sessions. Dropout rates (groups *with* vs *without* self-regulatory BCTs) were 29% vs 28%. The regression analysis revealed no effect of the self-regulatory BCTs on exercise adherence.

**Conclusion:**

An exercise adherence rate ≥50% for each FITT-principle and dropout rates at ~30% can be expected among patients taking part in long-term exercise interventions, combining resistance and endurance training during curative cancer treatment. Our results indicate that self-regulatory BCTs do not improve exercise adherence in interventions that provide evidence-based support to all participants (e.g., supervised group sessions).

**Trial registration:**

NCT02473003

## Introduction

Physical activity and exercise have proven safe and beneficial in patients undergoing cancer treatment, reducing several treatment-specific symptoms such as cancer-related fatigue^1-4^ and anxiety,^1,5^ as well as improving physical fitness and quality of life.^1,2,5^ Furthermore, epidemiological studies suggest that being physically active post-diagnosis may reduce the risk of cancer recurrence^6,7^ and improve overall survival.^7-9^ Although patients with cancer have much to gain from regular physical activity, the majority reduce their level of physical activity after being diagnosed^10-12^ due to disease symptoms, treatment side effects^13,14^ and insufficient support from healthcare.^15^

It is challenging for many patients to be physically active after a cancer diagnosis.^13-15^ However, recent reviews indicate that patients with cancer may benefit from behavioral interventions promoting physical activity and exercise.^16,17^ Such interventions typically involve strategies to facilitate exercise, including the use of behavior change techniques (BCTs), defined as active ingredients or practical components of an intervention designed to change a behavior.^18^ A taxonomy of 93 BCTs has been developed by Michie et al^18^ to provide a standardized method of classifying such intervention components. Using this taxonomy, exercise interventions involving patients with cancer often include self-regulatory BCTs such as goal-setting, self-monitoring, action planning, review of behavioral goals and problem solving.^16,17,19-21^ Several of these self-regulatory BCTs have been identified as having the potential to increase adherence to exercise interventions in patients with cancer.^16,17,19,20^ Exercise adherence in this context is defined as the extent to which intervention participants follow the exercise prescription (i.e. perform exercise according to the intervention protocol).^22,23^ A detailed description of adherence should include reports of adherence to frequency, intensity, time and type of exercise (FITT-principles).^24^ Other metrics such as dropout rates should also be included to provide insight into the efficacy and feasibility of exercise interventions.^23^

To date, adherence to exercise interventions in patients undergoing cancer treatment has been inadequately reported^16,24-26^ but is central to interpret study findings and implement effective exercise programs into clinical practice.^27^ Furthermore, although self-regulatory BCTs have frequently been used in interventions to facilitate exercise in patients undergoing cancer treatment,^16,20,21^ no clinical trial has directly examined their effect on exercise adherence according to the FITT-principles and dropout rates. This is of particular interest to better understand how to optimize behavioral support in exercise interventions for patients undergoing cancer treatment.

The aims of this study were therefore to (1) provide a detailed description of exercise adherence according to the FITT-principles and dropout rates, and (2) determine the effect of specific self-regulatory BCTs on exercise adherence in patients participating in a 6-month exercise intervention during curative cancer treatment. We hypothesized that self-regulatory BCTs would have a beneficial effect on exercise adherence in terms of higher adherence to the FITT-principles and lower dropout rates.

## Methods

### Study Design and Participants

This study was a secondary analysis using data from the Physical Training and Cancer (Phys-Can) study.^28^ The Phys-Can study is a multicentre randomized controlled trial (RCT) with a 2×2 factorial design (NCT02473003), approved by the Regional Ethical Review Board in Uppsala (Dnr 2014/249). All participants provided written informed consent to participate.

The Phys-Can study has previously been described in detail.^28^ Briefly, the aim of the Phys-Can study was to compare the effects of high (HI) versus low-to-moderate intensity (LMI) exercise *with* or *without* self-regulatory BCTs on cancer-related fatigue in patients undergoing curative (i.e. neoadjuvant or adjuvant) cancer treatment. Eligible patients were aged 18 years or over, newly diagnosed with breast, colorectal or prostate cancer and scheduled to undergo curative cancer treatment. Patients were excluded if they suffered from cognitive dysfunction (e.g., dementia and serious mental illness), physical impairments and/or other diseases (e.g., cardiovascular and lung diseases) that could affect their ability to perform exercise. The recruitment took place at University hospitals in three of the five largest cities in Sweden (Lund/Malmö, Linköping and Uppsala) between March 2015 and April 2018. The participants were randomized to one of four intervention groups: (1) HI *with* self-regulatory BCTs, (2) LMI *with* self-regulatory BCTs, (3) HI *without* self-regulatory BCTs or (4) LMI *without* self-regulatory BCTs.

### Intervention

The 6-month intervention started with a 6-week familiarization period and consisted of supervised group-based resistance training and home-based endurance training, *with* or *without* self-regulatory BCTs.^28^ The participants initiated the exercise programs during the same period as they started their cancer treatment. An overview of the exercise prescriptions used in the Phys-Can study is presented in Table 1. The exercise prescriptions were determined based on existing exercise guidelines and previous research in cancer populations.^29,30^ Exercise volume (intensity x time) was the same in all groups, with the HI groups exercising twice as intensely as the LMI groups but for half the time.

**Table 1.** Exercise Prescriptions According to the FITT-Principles in the Phys-Can Study.

	Principles	Exercise Prescriptions	
		Supervised Resistance Training	Home-Based Endurance Training
**HI**	**F**requency **I**ntensity **T**ime **T**ype	Two sessions/week Session 1 at 6RM and session 2 at 10RM Session 1 with 3×6 reps/exercise and session 2 with 3×10 reps/exercise. Maximum reps in the third set Seated leg press, chest press, leg extension, seated row, seated leg curl and seated overhead press	Two sessions/week 80-90% HRR 2 min intervals followed by 2 min of active rest. First 5 intervals (40 min/week), then 1 interval added every 4 weeks till max 10 intervals (80 min/week) Interval training
**LMI**	**F**requency **I**ntensity **T**ime **T**ype	Two sessions/week Session 1 at 50% of 6RM and session 2 at 50% of 10RM Session 1 with 3×12 reps/exercise and session 2 with 3×20 reps/exercise Seated leg press, chest press, leg extension, seated row, seated leg curl and seated overhead press	Not specified, spread throughout the week 40-50% of HRR 150 min/week, at least bouts of 10 min per session Continuous training

Abbreviations: FITT, Frequency, Intensity, Time and Type; Phys-Can, Physical Training and Cancer; HI, High intensity exercise; LMI, Low-to-moderate intensity exercise; RM, Repetition maximum; Reps, Repetitions; Min, minutes; HRR, Heart rate reserve.

#### Resistance training

The resistance training was offered twice per week in groups of 5 to 10 participants at public gyms and supervised by study coaches (physiotherapists and personal trainers). The program consisted of a total of six exercises (Table 1). To enable progression, all participants performed 6 and 10 repetitions maximum (RM) tests every 6 weeks, with subsequent adjustments made to their training programs.

#### Endurance training

The endurance training was home-based and followed up regularly by a coach at the gym. Participants in the HI groups were instructed to perform HI interval training (e.g., alternating between running and walking) twice per week. The total exercise time per week was 40 minutes (including active rest during the sessions) at the beginning of the training period, and was progressively increased up to 80 minutes at the end of the training period. Participants in the LMI groups were instructed to perform 150 minutes per week of LMI continuous training (e.g., bicycling or walking) in bouts of at least 10 minutes (Table 1). Individual heart rate reserve (HRR) was used to determine each participant’s target heart rate zone. HRR was calculated based on the results of a cardiopulmonary exercise test (CPET) performed before randomization where participants walked or ran on a treadmill until exhaustion.

#### Self-regulatory BCTs

The groups *with* self-regulatory BCTs were provided with goal-setting, self-monitoring, action planning, review of behavioral goals and problem solving. These BCTs were delivered face-to-face at nine occasions, except for self-monitoring that was performed by the participants after each training session. A detailed description of the specific self-regulatory BCTs used in the Phys-Can study is presented in Table 2.

**Table 2.** Self-Regulatory BCTs Provided in the Phys-Can Study.

BCTs	Description
Goal-setting	*Specific behavioral goals* for exercise performance were formulated by the participants in terms of exercise frequency, intensity, time and/or type. Goal-setting was regularly performed and the frequency was adjusted to individual needs.
Self-monitoring	Endurance and resistance training sessions were monitored and recorded by the participants in *training logs.* The logs included notes and reflections on situations when the participants actually performed the session, when they did not, and the subsequent consequences.
Action planning	Regular *exercise planning* specifying when, where and how to exercise were performed, based on interviews about previous exercise habits.
Review of behavioral goals	Behavioral goals were regularly reviewed to check if they were easily reached. *Adjustments* of goal-setting were made if the participant’s performance did not correspond to agreed goals.
Problem solving	*Analyses* of participants’ training logs with the coaches and identification of strategies to overcome barriers were performed concurrent with individual goal-setting and short-term action planning. A written *long-term coping planning* was developed at the end of the intervention, including strategies for overcoming barriers and using facilitators.

Abbreviations: BCTs, self-regulatory behavior change techniques; Phys-Can, Physical Training and Cancer.

#### Intervention integrity

All groups were provided with a certain level of support such as instructions on how to exercise, graded tasks, self-registration of exercise, feedback on exercise behavior as well as social support from coaches and peers. However, to ensure a clear delimitation between the conditions (i.e. *with* and *without* self-regulatory BCTs), several actions were taken. Before the intervention started, all coaches attended a 3-day mandatory course where they were trained to supervise participants randomized to one condition (i.e. either *with* or *without* self-regulatory BCTs). Coaches who provided self-regulatory BCTs supervised only participants randomized to this condition, and the groups trained on different days. Further, the coaches followed a detailed protocol during the intervention and kept a week-by-week checklist for each participant where notes about deviations from the protocol were made. Finally, research staff made repeated visits to the gyms and arranged group meetings (including twice-monthly tele conferences) with the coaches throughout the intervention to ensure that the intervention was delivered as planned.

### Measures

Data were collected between March 2015 and November 2018. Clinical and demographic information was gathered from medical records and study-specific forms. Exercise adherence, defined as the extent to which the participants followed the exercise prescription according to the intervention protocol,^22,23^ was assessed based on the FITT-principles and dropout rates. Overall adherence (i.e. total exercise volume) for resistance training and endurance training was also assessed. Exercise adherence was calculated as *performed training divided by prescribed training*. A detailed description of how each exercise adherence component was calculated and how adherence data were collected is presented in Table 3. For home-based endurance training, adherence to each FITT-principle was only calculated for the HI groups. Our study protocol did not enable these calculations for the LMI groups since the exercise prescription for frequency was not specified. Further, dropping out from the study was defined as a participant leaving the study or permanently discontinuing exercise before the end of the intervention, i.e. no training session performed during the last month or more. Participants who dropped out of the study were recorded as 0 adherence to any remaining training session until the end of the prescribed training period. Adherence data were collected from self-reports and objective instruments. For home-based endurance training, participants in the HI groups were asked to register any HI exercise (intervals and even continuous exercise if performed), while participants in the LMI groups were asked to register a maximum of 150 minutes of exercise at LMI as well as any HI exercise if performed.

**Table 3.** Calculation of Exercise Adherence and Data Collection in the Phys-Can Study.

Adherence	Groups	Calculation	Data Collection
**Supervised resistance training**			
Overall	HI and LMI	Total performed reps × sets × weight/ Total prescribed reps × sets × weight	Week-by-week checklists with attendance records maintained by the coaches; Records from 6 and 10RM tests results reported by the coaches; Resistance training logs recorded by the participants
Frequency	HI and LMI	Attended sessions/Prescribed sessions	
Intensity	HI and LMI	Performed weight/Prescribed weight during performed sessions	
Time	HI and LMI	Performed sets*repetitions/Prescribed sets*repetitions during performed sessions	
Type	HI and LMI	Sessions where all prescribed exercises were performed/Performed sessions	
**Home-based endurance training**			
Overall	HI and LMI	Total performed min at prescribed intensity**/Total prescribed min	Heart rate monitors; Study-specific endurance training logs recorded by the participants***
Frequency	HI*	Performed sessions/Prescribed sessions	
Intensity	HI*	Performed intervals where the target heart rate zone was met**/Prescribed intervals during performed sessions	
Time	HI*	Duration of performed intervals/Prescribed duration of intervals during performed sessions	
Type	HI*	Performed interval training sessions/Performed sessions	
**Dropout rates**			
	HI and LMI	Participants who left the study or did not exercise during the last month (or more) of the intervention/Participants	Week-by-week checklists with attendance records maintained by the coaches

Abbreviations: Phys-Can, Physical Training and Cancer; HI, High intensity; LMI, Low-to-moderate intensity; Min, minutes.

* Not applicable for the LMI groups.

** Prescribed intensity was adjusted as 70-90% HRR (HI groups) and 40% to 60% HRR (LMI groups) due to higher heart rates registered during chemotherapy treatment and lower heart rates registered during biking sessions despite adequate exertion level.

*** A combination of data from heart rate monitors and endurance training logs was used.

### Statistical Analyses

Exercise adherence is reported using descriptive statistics, i.e. mean percentage (SD) or number (%) as appropriate. Multiple linear regression (for resistance and endurance training separately) and logistic regression (for dropout rates) were performed to determine the effect of self-regulatory BCTs on each outcome. Analyses were conducted according to intention-to-treat and models were adjusted for exercise intensity, interaction (exercise intensity × self-regulatory BCTs), study site and cancer diagnosis. Dichotomous variables were generated for nominal variables with two categories (i.e. with/without BCTs, HI/LMI exercise and interaction of these variables). Study site and cancer diagnosis had three categories and were included in models using dummy-coding. The effect of the self-regulatory BCTs on each FITT-principle and dropout rates is presented as unstandardized regression coefficients (B) with 95% Confidence Intervals (95% CI). The regression coefficients can be interpreted as the mean difference in % adherence due to the main effect of the self-regulatory BCTs. All data analyses were performed with the Statistical Package for the Social Sciences (SPSS, v.25).

## Results

### Participants

In total, 600 participants were consecutively recruited. Twenty-three participants (4%) withdrew from the study before randomization and 577 (96%) were randomized to one of the four intervention groups. Participants’ mean age was 58.7 years (SD 12). The majority was diagnosed with breast cancer (n = 457), followed by prostate cancer (n = 97) and colorectal cancer (n = 23). Participants reported their previous exercise habits as median 210 minutes (IQR 120-360) of weekly moderate- to high-intensity exercise, and median 0 (IQR 0-1) weekly resistance training session. Participants’ characteristics in the four groups were similar at baseline (Table 4).

**Table 4.** Baseline Characteristics of All Randomized Participants in the Phys-Can Study.

	Groups with BCTs		Groups without BCTs	
	HI (n = 144)	LMI (n = 145)	HI (n = 144)	LMI (n = 144)
**Age** (years), mean (SD)	59 (13)	58 (12)	58 (11)	59 (12)
**Sex**, *n* (%)				
Male	29 (20)	27 (19)	28 (19)	28 (19)
Female	115 (80)	118 (81)	116 (81)	116 (81)
**Study site**, *n* (%)				
Malmö/Lund	65 (45)	68 (47)	66 (46)	64 (45)
Linköping	21 (15)	18 (12)	19 (13)	22 (15)
Uppsala	58 (40)	59 (41)	59 (41)	58 (40)
**Education**, *n* (%)				
Primary	16 (11)	12 (9)	14 (10)	20 (14)
Secondary	40 (28)	28 (20)	37 (27)	31 (22)
Tertiary	79 (56)	92 (66)	84 (61)	81 (58)
Other	6 (4)	8 (6)	3 (2)	7 (5)
**Occupation**, *n* (%)				
Working*	41 (30)	40 (29)	47 (35)	45 (33)
On sick leave	45 (32)	55 (39)	52 (39)	44 (32)
Retired	53 (38)	45 (32)	36 (27)	48 (35)
**Diagnosis**, *n* (%)				
Breast cancer	113 (78)	116 (80)	115 (80)	113 (79)
Prostate cancer	26 (18)	23 (16)	23 (16)	25 (17)
Colorectal cancer	5 (4)	6 (4)	6 (4)	6 (4)
Cancer treatment**, *n* (%)				
Chemotherapy	73 (51)	73 (50)	70 (49)	73 (51)
Radiation therapy	110 (76)	107 (74)	110 (76)	117 (81)
Endocrine therapy	85 (59)	94 (65)	88 (61)	94 (65)
**Comorbidities**, *n* (%)				
None	55 (44)	57 (45)	53 (42)	44 (35)
One or more	70 (56)	70 (55)	72 (58)	81 (65)
**Previous exercise habits**, median (IQR)				
Resistance training, times/week***	0 (0-1)	0 (0-1)	0 (0-1)	0 (0-0)
HI endurance training, min/week	0 (0-60)	0 (0-60)	0 (0-65)	0 (0-20)
LMI endurance training, min/week	180 (90-290)	200 (105-358)	150 (60-250)	150 (60-300)
Self-reported importance****, mean (SD)				
Resistance training	68 (29)	72 (29)	71 (30)	70 (29)
HI endurance training	56 (32)	60 (32)	56 (34)	54 (34)
LMI endurance training	77 (28)	79 (26)	76 (27)	79 (22)
**Exercise preferences**, n (%)				
HI intensity	66 (49)	77 (57)	70 (51)	64 (46)
LMI intensity	70 (51)	58 (43)	67 (49)	75 (54)

Abbreviations: Phys-Can, Physical Training and Cancer; HI, High intensity exercise; LMI, Low-to-moderate intensity exercise; BCTs, self-regulatory behavior change techniques; BMI, body mass index.

n’s do not all sum to total due to missing data; % is of those with available data.

* Full-time and part-time, not on any sick leave.

** Participants could have one type of treatment or a combination.

*** Reported on a 1 to 4 item scale (1 = “Not at all”, 2 = “Once a week”, 3 = “Twice a week”, 4 = Three times or more a week”).

**** Reported on a 100 mm visual analog scale anchored at “Not at all important” and “Very important”.

### Exercise Adherence

Missing data (two training logs in each group) were interpreted as no training sessions were performed (0% adherence). Exercise adherence to resistance and endurance training, as well as dropout rates, are provided for each intervention group in Table 5. The results below are reported in percentage (SD) for the groups *with* vs *without* self-regulatory BCTs respectively.

**Table 5.** Adherence to the Exercise Program in the Phys-Can Study.

	Groups with BCTs		Groups without BCTs	
Adherence	HI (n = 144)	LMI (n = 145)	HI (n = 144)	LMI (n = 144)
**Supervised resistance training** ^a^				
Overall (total volume)	48 (30)	53 (31)	49 (31)	52 (31)
Frequency (attendance)	50 (31)	54 (32)	52 (32)	54 (32)
Intensity (weight)	76 (37)	82 (37)	75 (38)	77 (40)
Time (sets and repetitions)	79 (38)	81 (37)	77 (38)	76 (40)
Type (exercises)	66 (43)	77 (39)	68 (42)	68 (43)
**Home-based endurance training** ^a^				
Overall (total volume)	39 (33)	58 (38)	42 (34)	51 (39)
Frequency (performed sessions)	47 (35)	-	51 (35)	-
Intensity (intervals at target heart rate)	57 (34)	-	62 (36)	-
Time (intervals at correct duration)	71 (36)	-	72 (38)	-
Type (interval training)	79 (36)	-	78 (38)	-
**Dropout rates** ^b^	45 (31)	41 (28)	45 (31)	36 (25)

Abbreviations: Phys-Can, Physical Training and Cancer; HI, High intensity exercise; LMI, Low-to-moderate intensity exercise; BCTs, self-regulatory behavior change techniques.

Data are ^a^mean percentage (SD) or ^b^number (%). Patients who dropped out of the study were recorded as 0 adherence to any remaining training session.

For supervised group-based resistance training (groups *with* vs *without* self-regulatory BCTs), participants performed on average 50% (31) and 51% (31) of the prescribed volume for the entire training period (overall adherence). More specifically, participants attended 52% (31) and 53% (32) of prescribed sessions. When participants attended a session, they performed 79% (37) and 76% (39) of prescribed intensity (weight), and 80% (37) and 77% (39) of prescribed time (set and repetitions). Finally, they adhered to exercise type in 71% (41) and 68% (42) of attended sessions (Table 5).

For home-based endurance training (groups *with* vs *without* self-regulatory BCTs), participants performed on average 48% (37) and 46% (36) of the prescribed volume for the entire training period (overall adherence). More specifically and only for the HI groups, participants performed on average 47% (35) and 51% (35) of prescribed sessions. They performed 57% (34) and 62% (36) of prescribed intensity, and 71% (36) and 72% (38) of prescribed time during the performed sessions. Finally, they adhered to exercise type in 79% (36) and 78% (38) of performed sessions (Table 5).

For dropout rates (groups *with* vs *without* self-regulatory BCTs), 29% and 28% of the participants permanently discontinued exercising during the intervention (Table 5).

### Effect of Self-Regulatory BCTs

The delivery of self-regulatory BCTs was similar between the groups allocated to receive this support (HI *with* BCTs: 39%, LMI *with* BCTs: 40%). The regression analysis revealed no effect of the self-regulatory BCTs on any outcome (Table 6). The main effects for supervised group-based resistance training ranged from −0.6 (95%CI [−5.7; 4.6], *P*-value = .83) for adherence to frequency to 3.1 (95%CI [−3.1; 9.2], *P*-value = .33) for adherence to time. The main effects for home-based endurance training ranged from −4.5 (95%CI [−12.4; 3.4], *P*-value = .27) for adherence to intensity to 1.9 (95%CI [−3.9; 7.6], *P*-value = .52) for overall adherence. Finally, the main effect for dropout rates was 0.08 (95%CI [−0.3; 0.4], *P*-value = .66).

**Table 6.** Main Effect of Self-Regulatory BCTs on Exercise Adherence in the Phys-Can Study.

Adherence	n	B (95%CI)^*^	*P*-Value
**Supervised resistance training**			
Overall	577	−0.6 (−5.6; 4.4)	.81
Frequency	577	−0.6 (−5.7; 4.6)	.83
Intensity	577	2.8 (−3.3; 8.9)	.37
Time	577	3.1 (−3.1; 9.2)	.33
Type	577	2.9 (−3.9; 9.7)	.40
**Home-based endurance training**			
Overall	577	1.9 (−3.9; 7.6)	.52
Frequency	288	−3.4 (−11.0; 4.5)	.40
Intensity	288	−4.5 (−12.4; 3.4)	.27
Time	288	−1.4 (−10.0; 7.1)	.74
Type	288	0.4 (−8.2; 9.0)	.92
**Dropout rates**	577	0.08 (−0.3; 0.4)	.66

Abbreviations: BCTs, Behavior change techniques; Phys-Can, Physical Training and Cancer; CI, Confidence Intervals.

Regression analyses were adjusted for exercise intensity, interaction (intensity × BCTs), study site and diagnosis when the four intervention groups were included in the analyses (=577). The analyses were adjusted for study site and diagnosis when the HI intervention groups only were included in the analyses (n = 288).

* Unstandardized coefficients represent the mean difference in adherence (%) due to the main effect of self-regulatory BCTs.

## Discussion

This study provides a comprehensive description of exercise adherence based on each FITT-principle and dropout rates in a large group of patients participating in a 6-month exercise intervention while undergoing curative cancer treatment. To our knowledge, this is the first study that directly examines the effect of self-regulatory BCTs on exercise adherence according to the FITT-principles and dropouts in patients during curative cancer treatment. We found an exercise adherence rate ≥50% for each FITT-principle and dropout rates at ~30%. Our results indicate that self-regulatory BCTs in the present setting did not improve exercise adherence.

For supervised group-based resistance training, exercise adherence was within the range reported in other exercise interventions involving patient undergoing curative cancer treatment.^27,31-34^ However, comparisons are limited due to the lack of studies reporting adherence according to all FITT-principles and the difference in calculation methods.^16,24-26^ Overall adherence and attendance were lower than adherence in terms of intensity, time and type. This demonstrates that attending training sessions twice a week during the entire intervention period was challenging for our participants, but when they did attend, they adhered to the prescribed program to a large extent. This also indicates that supervised group-based resistance training is an adequate form of training that may help patients to exercise according to prescribed intensity (weights) and time (sets and repetitions) while undergoing curative cancer treatment. However, the attendance rates were not optimal, which implies that encouraging patients to attend the gym may be the critical challenge for enhancing exercise adherence in future interventions. The difficulty of attending the gym may be explained by cancer-related barriers (e.g., disease and treatment-related symptoms, and medical appointments), which have been previously described as the main reason for lower attendance during treatment.^27,31,35,36^ Thus, our findings reflect the need to overcome such barriers where possible. A periodized exercise prescription with adjustments for such cancer-related barriers (e.g., an adapted progressive overload and recovery depending on treatment phase) could be appropriate. Indeed, one explanatory study reported that a “chemotherapy-periodization” of exercise for patients with breast cancer participating in supervised gym-based training resulted in higher attendance rates compared with regular exercise prescription.^31^ Similar adjustments could be made in future exercise interventions involving patients undergoing other cancer treatments, but research is needed to examine how such individualization could be applied. For home-based endurance training, frequency and intensity for HI training was lower in our study than previously reported in other intervention studies involving similar populations.^37-39^ Nonetheless, those interventions often consisted of supervised endurance training and/or fewer intervals, which could explain the observed differences. The dropout rates within the Phys-Can study were higher than those reported in previous studies involving patients with cancer.^40^ However, those interventions were usually shorter, conducted after treatment and involved less comprehensive exercise programs, therefore taking part in our intervention was likely more challenging for participants.^40^ Another possible explanation is that we included in our calculations of dropout rates participants who permanently discontinued exercise before the end of the intervention without leaving the study. Studies usually only include participants who did not complete follow-up assessments.^34,37,41-43^ However, we believe that including participants who permanently discontinued exercise gives a more accurate picture of the feasibility of such exercise programs during curative treatment,^23^ which is valuable information for future interventions.

In the present study, we used a combination of five frequently used self-regulatory BCTs (goal-setting, self-monitoring, action planning, review of behavioral goals and problem solving) to facilitate exercise in two of the four intervention groups. The analyses did not reveal any effect of these specific BCTs on any aspect of exercise adherence (FITT-principles and dropout rates). Our results are in contrast with previous studies, where self-regulatory BCTs were found to be associated with higher adherence to exercise interventions in patients with cancer.^17,20^ However, it is important to take into account that all groups in the Phys-Can study were provided with evidence-based support such as supervision, instructions on how to exercise, graded tasks, self-registration of exercise, feedback on exercise behavior as well as social support from coaches and peers.^16,17,19,21^ This was done to ensure that all participants achieved a minimum exercise volume in order to detect possible effects of exercise intensity on the Phys-Can study’s main outcome. It was a precarious balance between being able to evaluate the effects of exercise on the main outcome and evaluating the effect of self-regulatory BCTs on exercise adherence. Thus, the lack of effect of the self-regulatory BCTs could be due to the fact that all groups were provided with enough support to facilitate exercise, and that self-regulatory BCTs as additional support was not sufficient to make a difference. This is consistent with findings published in a previous mixed-methods study, where our participants described that they perceived the support provided to all as more useful than self-regulatory BCTs for performing exercise.^44^ The lack of effect of the self-regulatory BCTs could also be explained by our participants’ motivation level and exercise habits. All participants agreed to take part in a demanding exercise intervention for 6 months during cancer treatment and so were per definition motivated enough to participate. Further, a majority was already physically active before entering the study. Previous research has demonstrated that baseline motivation and previous exercise habits are strong predictors for exercise adherence after a cancer diagnosis.^45,46^ It is thus possible that a more heterogeneous clinical population with lower baseline motivation levels and poorer exercise habits may benefit from these self-regulatory BCTs, but this needs to be further investigated. Finally, another possible explanation is that, despite a high motivation to participate in the study and the use of self-regulatory BCTs, participants did not manage to exercise as prescribed because the prescription may not have been totally in line with their own exercise preferences and needs. Our participants were randomized to one condition and could therefore not choose to perform the exercise mode (resistance vs endurance) and intensity (LMI vs HI) they preferred. Yet it is well established that incorporating patients’ preferences and determining what is important to them is essential for behavioral strategies to be effective.^47^

The strengths of our study include using data from a large multicentre RCT to provide detailed information about exercise adherence and effect of self-regulatory BCTs in patients undergoing curative cancer treatment. Intervention integrity was carefully controlled to ensure a clear delimitation between the conditions (i.e. *with* and *without* self-regulatory BCTs). A further strength is the detailed description of the study exercise prescription and adherence, allowing replication of our intervention and highlighting the difficulties that patients may encounter. Limitations include the risk that participants may have under- or over reported endurance training sessions during the 6-month intervention or had technical issues with the heart rate monitors, which could affect the descriptive data. However, by using a combination of data from training logs and heart rate monitors, most of the performed sessions were correctly captured. Furthermore, we could not calculate adherence to frequency, intensity, time, and type for endurance training in the LMI groups. This is because no exercise prescription for frequency was determined by our study protocol. Further, participants in the LMI groups were asked to report or record a maximum of 150 minutes of LMI per week (and all HI exercise) to minimize burden that a self-registration of exercise for 6 months may imply. We were therefore limited in the possibilities to explore this data in depth. However, the overall adherence rates calculated (corresponding to the exercise volume performed during the entire intervention) still provide valuable information for the LMI groups. Finally, the generalizability of our findings may be limited, considering that our participants were mainly women treated for breast cancer, who were physically active, highly educated and motivated to take part in a comprehensive 6-month intervention. Thus, our results may not be applicable to all patients in clinical settings. However, readers are provided with details regarding the study settings and baseline characteristics of our sample, and should be able to evaluate for which target groups the study provides valuable information.

## Conclusion

An exercise adherence rate at ≥50% for each FITT-principle and dropout rates at ~30% can be expected among patients taking part in long-term exercise interventions, combining supervised group-based resistance training and home-based endurance training during curative cancer treatment. Our results indicate that specific self-regulatory BCTs do not improve exercise adherence in comprehensive interventions that provide evidence-based support to all participants (e.g., supervised group sessions). Thus, such support may be sufficient for patients motivated enough to exercise during treatment, which is worth considering when aiming to implement exercise interventions in clinical settings. However, a more heterogeneous population with low motivation and exercise levels may benefit from these self-regulatory BCTs, but this needs to be further investigated.
